# The Tunnel Window Sign Identifies Active Scabies Infestation: A Validation Study

**DOI:** 10.3390/jcm15186972

**Published:** 2026-09-09

**Authors:** Mateusz Krzysztof Mateuszczyk, Pablo Moreno-Reolid, Magdalena Łyko, Joanna Maj

**Affiliations:** 1 University Centre of General Dermatology and Oncodermatology, Faculty of Medicine, Wroclaw Medical University, 50-556 Wroclaw, Poland; 2 Department of Applied Physics, University of Granada, 18071 Granada, Spain

**Keywords:** scabies, dermoscopy, tunnel window sign, *Sarcoptes scabiei*, ultraviolet-induced fluorescence dermoscopy, treatment monitoring

## Abstract

**Background/Objectives:** Scabies, caused by *Sarcoptes scabiei*, is a recognised neglected disease of rising global burden, and increasing resistance to first-line scabicides is making treatment failure more frequent. No dermoscopic marker of cure has been validated for monitoring treatment response, so a reliable marker of active infestation is crucial. The tunnel window sign (TWS)—rounded perforations along the burrow, visible under polarised dermoscopy and ultraviolet-induced fluorescence dermoscopy (UVFD)—is a recently introduced candidate. We aimed to determine whether TWS represents a dermoscopic marker of active scabies infestation by evaluating its specificity, treatment dynamics and diagnostic performance under polarised dermoscopy and UVFD. **Methods:** In this single-centre observational study conducted in 2026, 33 patients with active scabies (Cohort 1; 106 burrows in 30 permethrin–ivermectin-adherent and 11 in 3 non-adherent) and 30 controls with excoriated pruritic dermatoses (Cohort 2; 150 lesions) were examined. All Cohort 1 patients were re-evaluated every 4 weeks until resolution. Paired polarised/UVFD images were re-scored by four raters (three blinded). **Results:** TWS was undetectable in all 30 controls with pruritic dermatoses and in all 30 post-treatment adherent patients (0/30 and 0/30, respectively). It resolved with effective treatment (undetectable from week 4; McNemar *p* < 0.0001), preceding pruritus resolution, and persisted in non-adherent patients until retreatment. At the burrow level, UVFD detected TWS more sensitively than polarised dermoscopy (68/106 vs. 60/106; *p* = 0.0078; adjusted OR 1.38, 95% CI 1.07–1.79). Inter-rater agreement was almost-perfect (Fleiss’ κ = 0.872). **Conclusions:** TWS behaves as a dermoscopic marker of active scabies infestation: it disappears after successful treatment, persists following treatment failure, and distinguishes active infestation from both post-treatment skin and non-scabietic pruritic dermatoses. UVFD improves its detection compared with polarised dermoscopy.

## 1. Introduction

Scabies, caused by infestation with *Sarcoptes scabiei* var. hominis, is among the most prevalent skin diseases worldwide and a re-emerging concern [1,2]. Definitive diagnosis under the 2020 International Alliance for the Control of Scabies (IACS) criteria requires the mite or its products to be directly visualised [3]. Dermoscopy has progressively replaced light microscopy as the first-line bedside technique, owing to its non-invasiveness, equivalent or superior accuracy, and ease of use [4]. Recent comprehensive reviews summarise these diagnostic advances—including ultraviolet-induced fluorescence dermoscopy—alongside current and emerging treatments for scabies [5]. First-line treatment relies on topical 5% permethrin and oral ivermectin, with benzyl benzoate as an alternative; a recent national multicentre study found permethrin substantially less effective than benzyl benzoate or ivermectin (cure rate 42.5% vs. 84.2% and 81.6%), while environmental decontamination counselling was associated with reduced treatment failure [6]. In response to these limitations, newer scabicides are being introduced. These include topical spinosad 0.9%, a single-application agent effective with minimal systemic absorption and recently approved for scabies, and—most notably—oral moxidectin, a macrocyclic lactone whose extended half-life (~23 days) may cover the entire life cycle of *Sarcoptes scabiei* in a single dose, with superior skin penetration and superior efficacy to ivermectin in preclinical models and which is now under clinical evaluation [7]. Together, these developments underscore the value of an objective, non-invasive marker of persisting active scabies for confirming treatment response across evolving regimens.

A growing taxonomy of dermoscopic signs has accompanied this shift, including the pathognomonic “triangle” sign [8], the “grey-edged line” of melanised faecal material [9], the “wake sign” of trailing scales [10], and the “noodle pattern” of crusted scabies [11]. Ultraviolet-induced fluorescence dermoscopy (UVFD, typically 365 nm) adds further information: burrows fluoresce as bright bluish-white tracts, while mites and eggs may appear as greenish “ball” structures [12,13,14,15]. A recent multicentre study found UVFD and polarised dermoscopy to be complementary, with UVFD better detecting burrows and eggs, particularly in skin of colour [16,17].

We recently introduced the term tunnel window sign (TWS) to describe rounded perforations along an active scabies burrow, particularly apparent under UVFD [18]. Our first report was a small proof-of-concept series (10 patients) providing only preliminary specificity data (excoriation controls), without a formal modality comparison or any longitudinal follow-up, leaving the diagnostic performance and treatment dynamics of TWS unvalidated [18]. Here we report a paired-modality study in 33 consecutive patients with active scabies, with two complementary control cohorts (excoriated pruritic dermatoses and post-treatment scabies). Our objectives were (i) to test whether UVFD detects TWS more sensitively than polarised dermoscopy and (ii) to evaluate whether TWS discriminates active scabies from alternative pruritic dermatoses and from successfully treated scabies, supporting its role as a marker of active infestation.

## 2. Materials and Methods

### 2.1. Study Design and Setting

This was a single-centre, observational study (STROBE-compliant) conducted at the University Centre of General Dermatology and Oncodermatology, Wroclaw Medical University, Wrocław, Poland, in 2026. Recruitment took place between January and April 2026. The study was observational and was not registered; the institutional Bioethics Committee determined that it did not constitute a medical experiment (certificate no. 306/2026). No formal sample-size calculation was performed; a consecutive convenience sample of all eligible patients presenting during the study period was analysed. Patients were prospectively examined and photographed as part of routine care, with dermoscopic findings subsequently analysed. The study was conducted in accordance with the Declaration of Helsinki. All participants (or legal guardians for participants < 16 years; jointly for those aged 16–17) provided written informed consent.

### 2.2. Participants

Cohort 1—Active scabies. Thirty-three consecutive patients meeting the 2020 IACS criteria for confirmed scabies (Level A3: mite visualised on dermoscopy) were prospectively enrolled [3]. Exclusion criteria comprised previous scabicidal therapy, crusted scabies, and pregnancy or breastfeeding. Of these, 30 patients adhered to the prescribed regimen and constituted the per-protocol baseline (106 burrows imaged); 3 patients with documented non-adherence at week 4 were analysed separately as a non-adherent subgroup (11 additional burrows imaged).

Cohort 2—Pruritic dermatoses (specificity controls). A total of 30 patients with excoriated pruritic dermatoses were recruited consecutively at the same centre during the same period: 20 with atopic dermatitis (Hanifin–Rajka criteria) [19], 5 with contact dermatitis, and 5 with pruritic xerosis. Patients with concomitant scabies or recent scabicidal treatment were excluded. These conditions were selected as specificity controls because they represent the principal pruritic, frequently excoriated differential diagnoses of scabies, providing a stringent test of TWS specificity.

Cohort 3—Post-treatment scabies (dynamic controls). All 33 Cohort 1 patients were re-evaluated every 4 weeks until resolution, defined clinically as the complete absence of cutaneous lesions on whole-body examination and absence of pruritus. The tunnel window sign was not used as a criterion for declaring cure. All patients were prescribed a two-dose regimen of topical 5% permethrin cream and oral ivermectin (200 µg/kg) at baseline and 2 weeks later, per the 2021 CDC guidance [20], 2017 European guidelines [21] and the respective Summary of Product Characteristics; concurrent environmental decontamination was recommended [20,21]. Environmental decontamination followed standardised written and verbal instructions given at baseline such as machine washing of clothing, bed linen and towels at 60 °C and sealing of non-washable items in plastic bags for at least 72 h. All household members and sexual partners were treated simultaneously, irrespective of symptoms [21]. Patients with persistent disease at follow-up were re-counselled on adherence and offered the full regimen again. Pruritus was assessed using the Numerical Rating Scale (NRS, 0–10) [22] at each visit. Adherence to the prescribed regimen was assessed at the start of each visit by structured interview, before the clinical and dermoscopic examination, and by confirmation of concurrent treatment of household members and contacts; no pharmacy-dispensing or medication-count verification was performed. The three non-adherent patients disclosed non-adherence during this pre-examination interview.

### 2.3. Dermoscopic Examination

All images were acquired with a DermLite DL5 dermatoscope (3Gen, San Juan Capistrano, CA, USA) at ×10 magnification, paired with a smartphone camera adapter, by a single dermatologist. For every identified burrow, paired images were obtained sequentially: one under polarised contact dermoscopy and one under UVFD (365 nm). In Cohort 2, exactly 5 randomly selected excoriations per patient (*n* = 150 lesions) were imaged with both modalities. In Cohort 3, the same anatomical sites imaged at baseline were re-imaged, with a full-body clinical and dermoscopic assessment at each follow-up. At interdigital and finger-web sites, the fingers were abducted and the web stretched, with the dermatoscope faceplate applied tangentially to the flattened skin; where firm contact could not be achieved, non-contact polarised and UV examination at a short working distance was used instead.

### 2.4. Definition and Scoring of the Tunnel Window Sign

**Figure 1:**
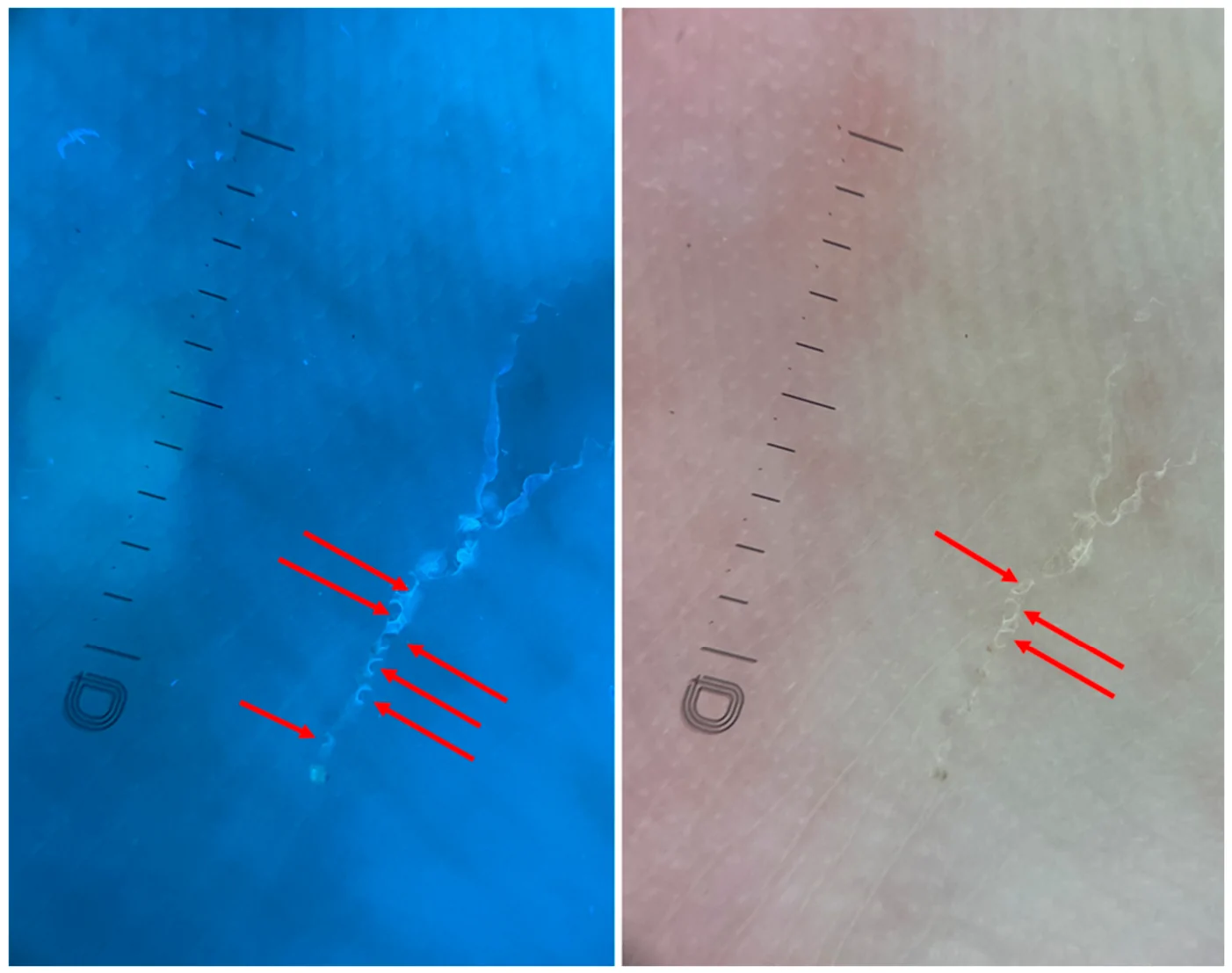
TWS in active scabies. Representative burrow under ultraviolet-induced fluorescence dermoscopy (UVFD) (**left panel**) and polarised dermoscopy (**right panel**); rounded openings (“windows”) distributed along the course of the burrow, appearing bright blue under UVFD. DermLite DL5, ×10. Red arrows indicate the tunnel window sign. The dark linear marks represent the dermatoscope’s built-in measurement scale.

TWS was defined, consistent with our previous publications [18,23], as rounded openings distributed along the course of a scabies burrow, visible under both polarised dermoscopy and UVFD (where they appear bright blue) (Figure 1). Presence was recorded as binary (present/absent) per individual burrow in each modality. Image scoring was conducted independently by four raters of varying expertise: the principal investigator (who originally described the sign; M.K.M.), a dermoscopy expert (M.Ł.), a dermatologist without dermoscopy experience, and a non-medical observer. Paired images were anonymised and evaluated in randomised order, with all raters blinded to one another’s assessments; the three additional raters were also blinded to clinical context and cohort assignment, whereas the principal investigator, who had acquired the images and originally described the sign, could not be blinded to clinical context.

### 2.5. Unit of Analysis and Statistical Analysis

The primary unit of analysis was the individual burrow (mite-level); a secondary patient-level analysis classified a patient as TWS-positive in a given modality if ≥1 burrow showed the sign. Categorical variables are reported as counts and Wilson 95% confidence intervals (CI). The primary analysis compared TWS detection between modalities in the per-protocol Cohort 1 (*n* = 106 burrows from 30 adherent patients) using McNemar’s exact test. To account for clustering of burrows within patients, a generalised estimating equations (GEE) model with a logit link and exchangeable working correlation was fitted as a sensitivity analysis. Sensitivity, specificity, and predictive values were calculated at mite and patient levels. Inter-rater agreement was quantified using Cohen’s kappa (pairwise) and Fleiss’ kappa (overall), interpreted according to Landis and Koch [24]. A two-sided *p* < 0.05 was considered significant. The GEE model was fitted in R 4.6.0 (geepack 1.3.13); all other analyses were performed in Python 3.11.15 (scipy 1.17.1). Non-adherent patients were analysed descriptively as a separate subgroup; no formal between-modality test was performed given the small sample size. Reporting follows STROBE [25] and STARD [26] where applicable. The anatomical-site comparison was exploratory and post hoc. All 33 patients completed follow-up to resolution; there was no loss to follow-up and no missing index-test data.

## 3. Results

### 3.1. Cohort Characteristics

A total of 63 participants were included (Table 1; participant flow in Figure S1). Cohort 1 comprised 33 patients with active scabies (19 men, 14 women; age range 14–75 years; Fitzpatrick I–II); 30 adhered to treatment and constituted per-protocol Cohort 3, while 3 with documented non-adherence at week 4 were analysed separately. Cohort 2 comprised 30 patients with excoriated pruritic dermatoses (15 men, 15 women; age range 12–45 years; Fitzpatrick I–II). At the end of follow-up, all 33 scabies patients were classified as cured. No treatment-related adverse events were recorded.

### 3.2. Burrow Inventory and Anatomical Distribution

**Figure 2:**
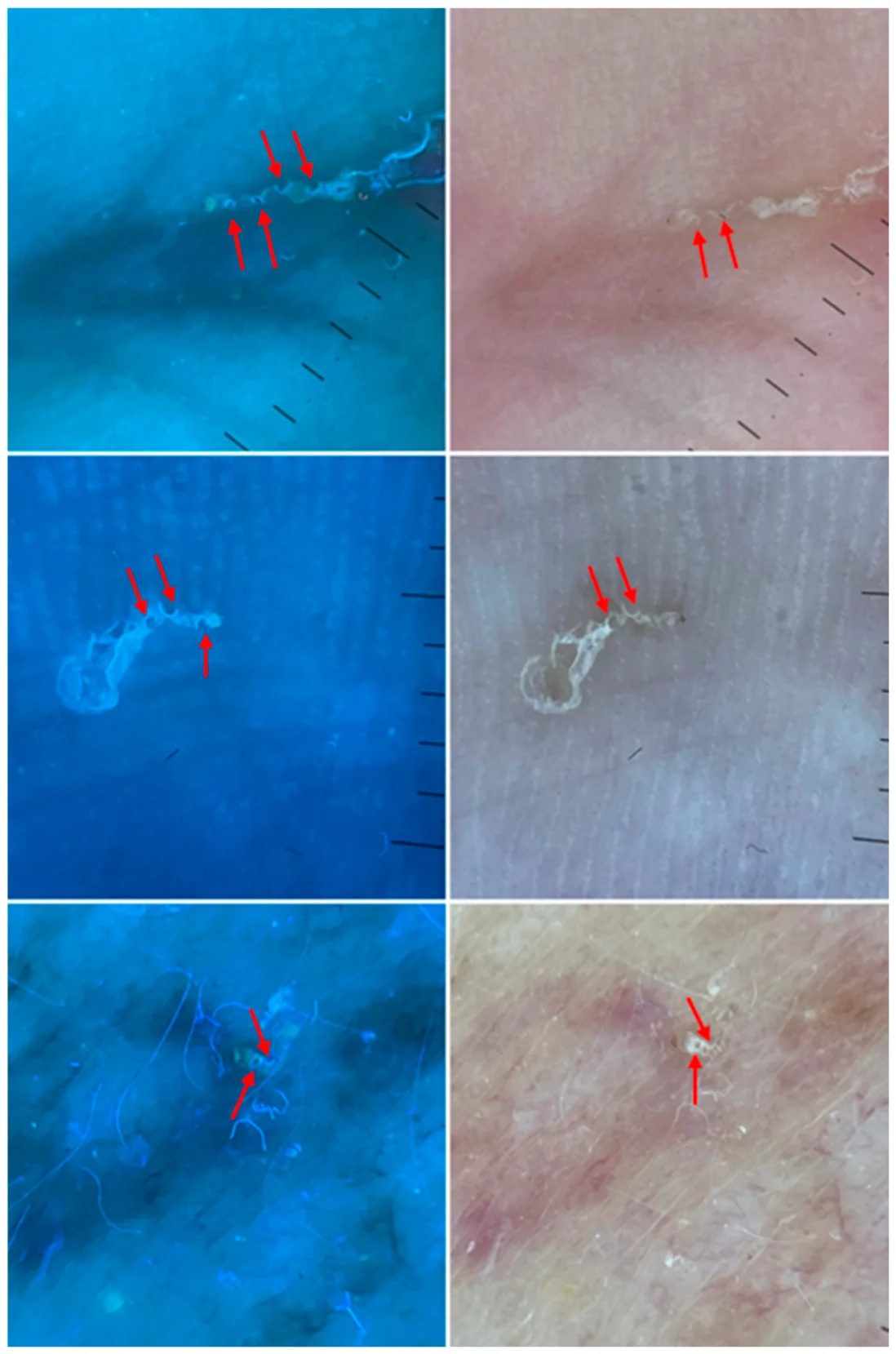
TWS across anatomical sites. Representative TWS-positive burrows on the hand (**upper panels**), foot (**middle panels**), and trunk (**lower panels**), which are the three sites imaged in the per-protocol cohort. DermLite DL5, ×10. Red arrows indicate the tunnel window sign. The dark linear marks represent the dermatoscope’s built-in measurement scale.

A total of 106 burrows were identified across the 30 adherent Cohort 1 patients, yielding 212 paired observations (106 polarised + 106 UVFD); 11 additional burrows were imaged in the 3 non-adherent patients (described separately under “Treatment kinetics in initially non-adherent patients”). The median number of imaged burrows per adherent patient was 3 (range 1–10). Anatomical distribution in the per-protocol analysis: hands 52/106 (49.1%), trunk 41/106 (38.7%), feet 13/106 (12.3%) (Figure 2). No facial involvement was observed in the per-protocol cohort.

### 3.3. Primary Analysis—Paired Comparison of TWS Detection

**Figure 3:**
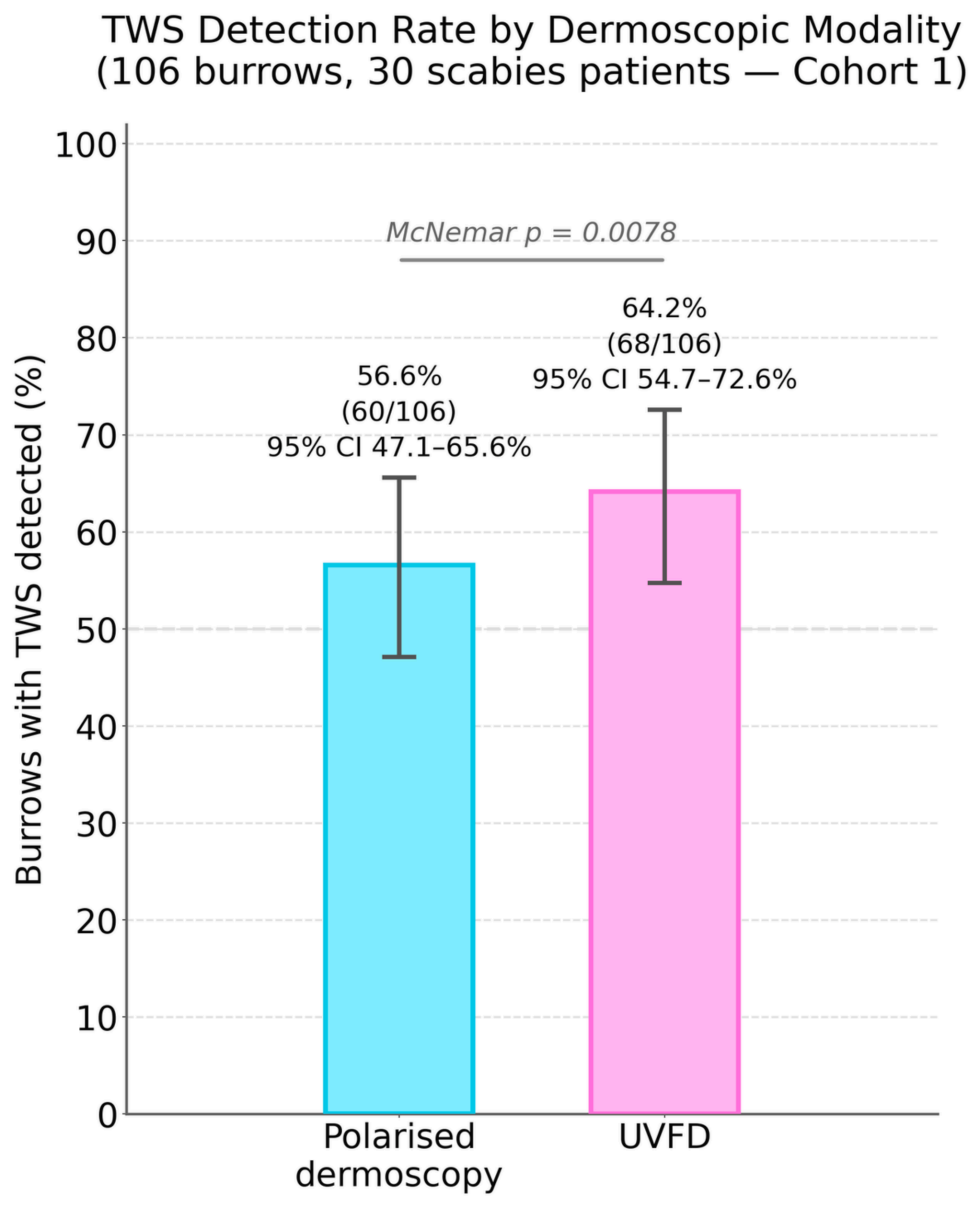
TWS detection rate by dermoscopic modality. Per-burrow TWS detection in Cohort 1 (106 burrows, 30 patients). Error bars represent 95% Wilson confidence intervals.

**Figure 4:**
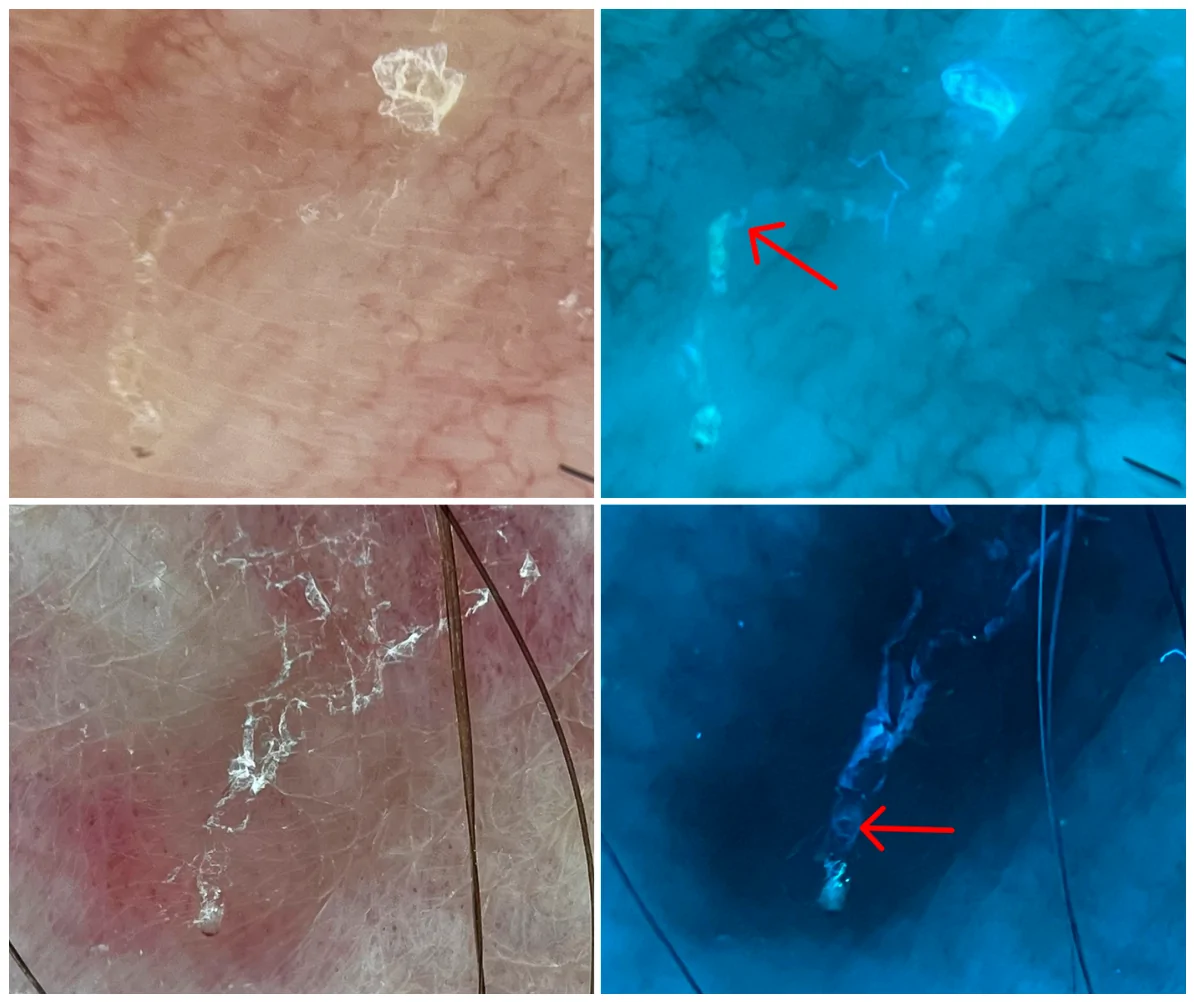
Modality-dependent visibility of TWS. Arrows mark the tunnel window sign, visible as bright-blue rounded openings under UVFD (**right panels**) but not observed under polarised dermoscopy (**left panels**). DermLite DL5, ×10. The dark linear marks represent the dermatoscope’s built-in measurement scale.

TWS was detected in 60/106 burrows (95% CI 47.1–65.6) under polarised dermoscopy and in 68/106 burrows (95% CI 54.7–72.6) under UVFD (Figure 3). UVFD-identified TWS in 8 burrows scored negative under polarised dermoscopy, whereas polarised dermoscopy did not identify TWS in any burrow scored negative under UVFD (Figure 4). McNemar’s exact binomial test confirmed a significant difference between modalities (*p* = 0.0078), corroborated by the chi-square approximation with Yates’s correction (χ^2^ = 6.13, *p* = 0.013). The advantage of UVFD remained significant in the GEE sensitivity analysis accounting for within-patient clustering (adjusted OR 1.38, 95% CI 1.07–1.79; *p* = 0.012), with substantial intraclass correlation (α = 0.40).

### 3.4. Anatomical Subgroup Analysis

**Figure 5:**
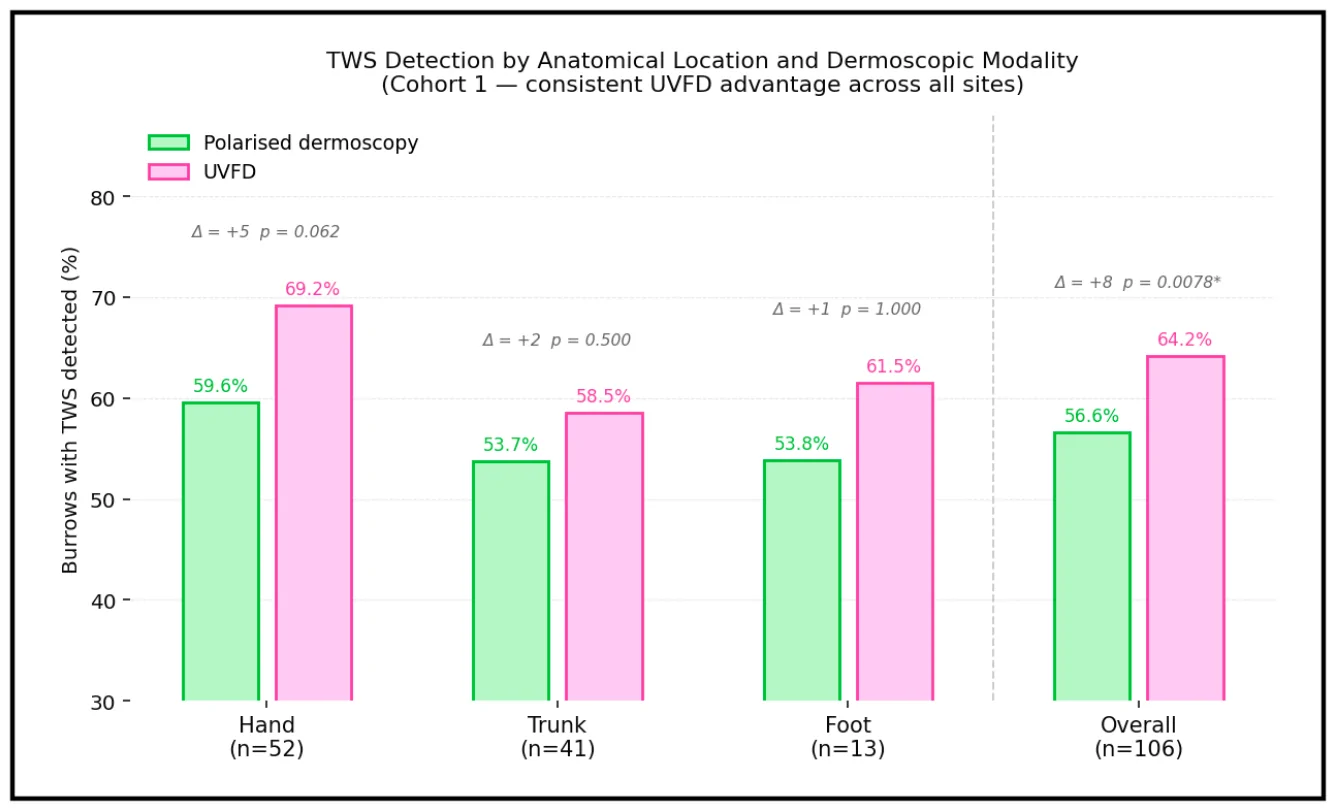
TWS detection by anatomical site and dermoscopic modality. UVFD showed a consistent advantage over polarised dermoscopy across all three sites. Δ = difference in number of TWS-positive burrows (UVFD minus polarised). The dashed vertical line separates the individual anatomical sites from the overall (pooled) comparison. * *p* < 0.05 (McNemar’s exact test).

UVFD consistently detected TWS in more burrows than polarised dermoscopy across all sites, although none of the site-specific comparisons reached significance (Figure 5). The largest absolute UVFD advantage was on the hands (5 additional cases, *p* = 0.062). The consistent positive UVFD advantage across all sites (Δ = +5 hands, +2 trunk, +1 feet) supports a generalised modality effect rather than a site-specific phenomenon. The hand subgroup, accounting for nearly half of all imaged burrows (52/106), represents the most clinically relevant site, as scabies preferentially affects the interdigital spaces, wrists, and palms [27]. Per-patient detection rates were 93% on hands (13/14), 93% on trunk (14/15), and all on feet (4/4).

### 3.5. Inter-Rater Agreement

**Figure 6:**
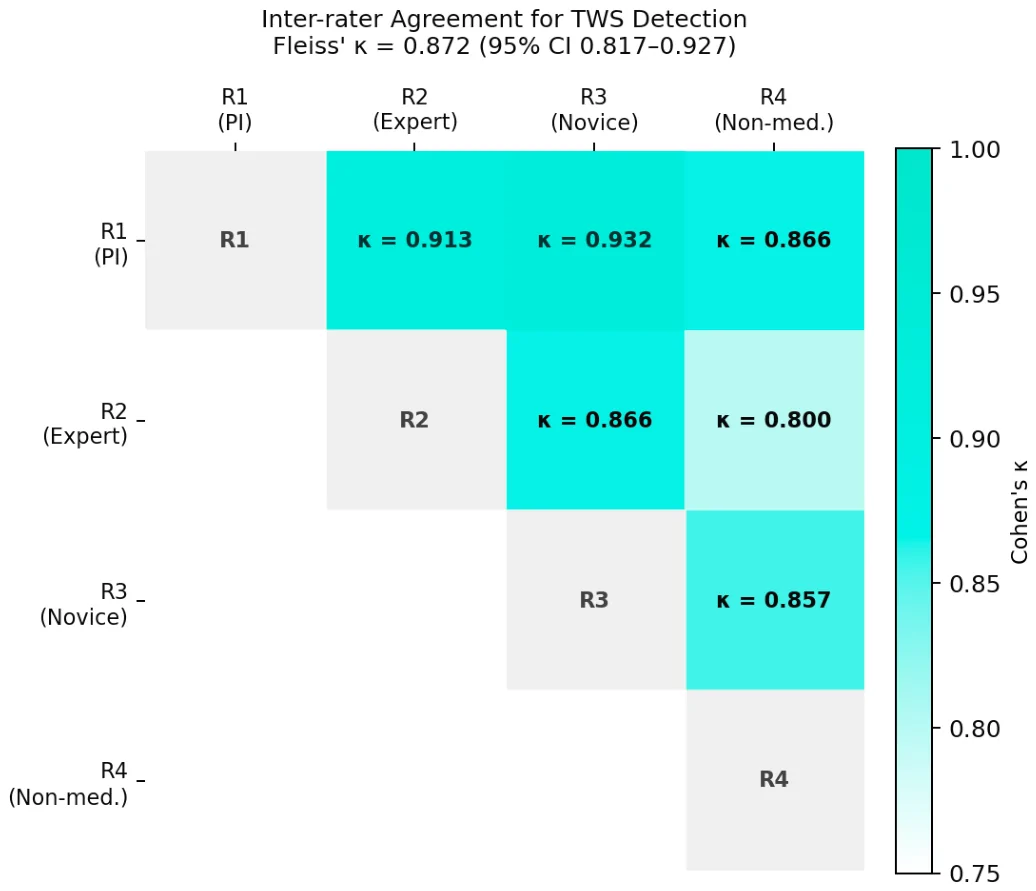
Inter-rater agreement for TWS detection. Pairwise Cohen’s κ heatmap across four raters of varying expertise (R1: principal investigator (M.K.M.); R2: dermoscopy expert (M.Ł.); R3: novice dermatologist; R4: non-medical observer) Images were anonymised and scored in randomised order and blinded to one another’s assessments; R2–R4 were additionally blinded to clinical context, whereas R1 could not be blinded, having originally described the sign.

Raters received the original TWS publication [18] as standardised training. Overall agreement across all four raters was almost perfect (Fleiss’ κ = 0.872, 95% CI 0.817–0.927); pairwise Cohen’s κ ranged from 0.800 to 0.932 (Figure 6). Full four-rater concordance was observed for 186/212 images (87.7%). All discordances between the principal investigator and other raters were unidirectional (PI-positive/rater-negative). This may reflect a more sensitive detection threshold; however, because the principal investigator was unblinded to clinical context and had originally described the sign, an element of observer (confirmation) bias cannot be excluded, and this is acknowledged as a limitation. Notably, agreement among the three independent raters alone remained high (pairwise κ 0.80–0.87), indicating that the reproducibility of TWS was maintained even without the principal investigator’s assessments. In a sensitivity analysis restricted to the three raters with no role in image acquisition or enrolment (R2–R4, majority vote), the between-modality effect was preserved: TWS was detected in 67/106 burrows under UVFD versus 58/106 under polarised dermoscopy (McNemar exact *p* = 0.0039), with no burrow positive under polarised light alone, and patient-level UVFD sensitivity remained 30/30. The primary finding is therefore not dependent on the principal investigator’s readings.

### 3.6. Specificity Controls—Cohorts 2 and 3

**Figure 7:**
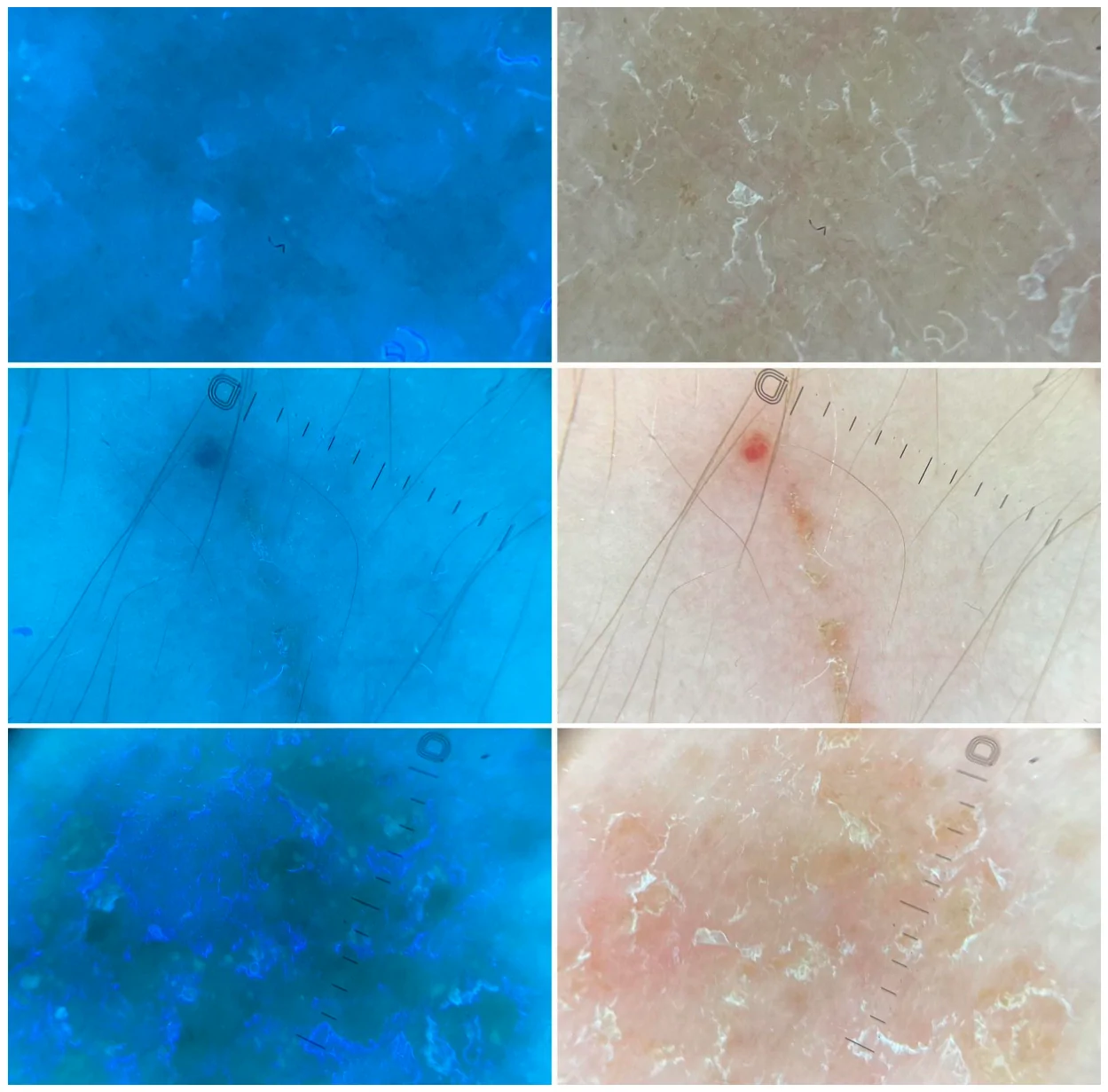
Absence of TWS in pruritic dermatoses (specificity controls) under polarised dermoscopy (**right panels**) and UVFD (**left panels**): pruritic xerosis (**upper panels**), atopic dermatitis (**middle panels**), and contact dermatitis (**lower panels**). DermLite DL5, ×10. No rounded openings (tunnel windows) are present in any panel. The dark linear marks represent the dermatoscope’s built-in measurement scale.

Across 30 patients with excoriated pruritic dermatoses and 150 imaged lesions (Cohort 2), TWS was not identified in any lesion under either modality (0/30 patients TWS-positive; 0/20 atopic dermatitis, 0/5 contact dermatitis, 0/5 pruritic xerosis) (Figure 7). At the 4-week follow-up of adherent scabies patients (Cohort 3), TWS was not detected in any patient (0/30).

### 3.7. Patient-Level Diagnostic Performance

Using TWS positivity in ≥1 burrow under UVFD as the diagnostic test, with active scabies (Cohort 1 baseline, *n* = 30 adherent) versus the combined non-active group (Cohort 2 + adherent Cohort 3 at week 4, *n* = 60) as the reference, UVFD yielded 30 true positives, 0 false negatives, 60 true negatives, and 0 false positives, with sensitivity 30/30 (95% CI 88.6–100.0), specificity 30/30 in pruritic-dermatoses controls (95% CI 88.6–100.0), and 30/30 in post-treatment patients (95% CI 88.6–100.0), with a pooled 60/60 (95% CI 94.0–100.0) as a combined summary of accuracy 90/90 (95% CI 95.9–100.0). The corresponding values for polarised dermoscopy were sensitivity 28/30 (93.3%), specificity 60/60, accuracy 88/90. The comparison of TWS prevalence between active scabies and combined controls was highly significant (Fisher’s exact *p* < 0.0001).

### 3.8. Dynamic Behaviour of TWS During Treatment (Adherent Patients)

**Figure 8:**
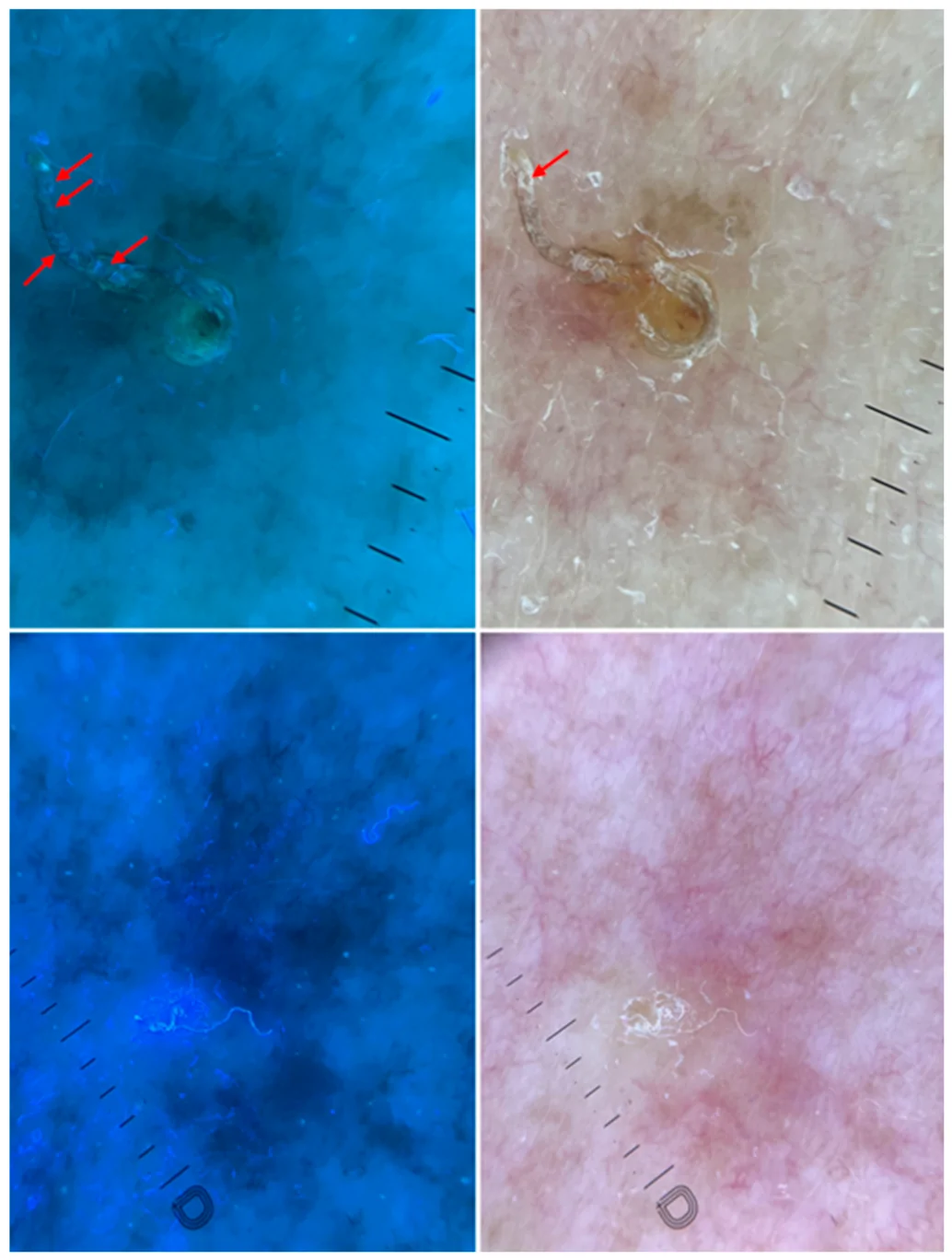
Resolution of TWS after treatment. The same anatomical site at baseline (**upper panels**) and at the week-4 reassessment after permethrin–ivermectin therapy (**lower panels**); the tunnel window sign present at baseline is no longer detectable. DermLite DL5, ×10. Red arrows indicate the tunnel window sign. The dark linear marks represent the dermatoscope’s built-in measurement scale.

All 30 adherent patients exhibited TWS in ≥1 burrow under UVFD at baseline. At the 4-week reassessment, TWS was no longer detectable in any of the 30 adherent patients under either modality (0/30; McNemar exact *p* < 0.0001) (Figure 8). At the 8-week reassessment, all 30 adherent patients were classified as cured based on complete absence of cutaneous lesions and pruritus. At baseline, all 30 adherent patients reported pruritus (mean NRS 7/10); at week 4, despite the complete disappearance of TWS, 6/30 (20%) still reported pruritus (individual NRS scores: 3, 4, 4, 6, 7, 8; median 5, mean 5.3); by week 8, no patient reported any pruritus (NRS 0/10 in all 30/30). The dissociation between early TWS disappearance at 4 weeks and the persistence of pruritus in a subset of patients—with full clinical resolution by 8 weeks—supports TWS as an early marker of treatment response: residual pruritus after successful treatment reflects a persistent immune–inflammatory response [28] and is a well-documented clinical phenomenon (post-scabies pruritus) [29].

### 3.9. Treatment Kinetics in Initially Non-Adherent Patients (n = 3)

**Figure 9:**
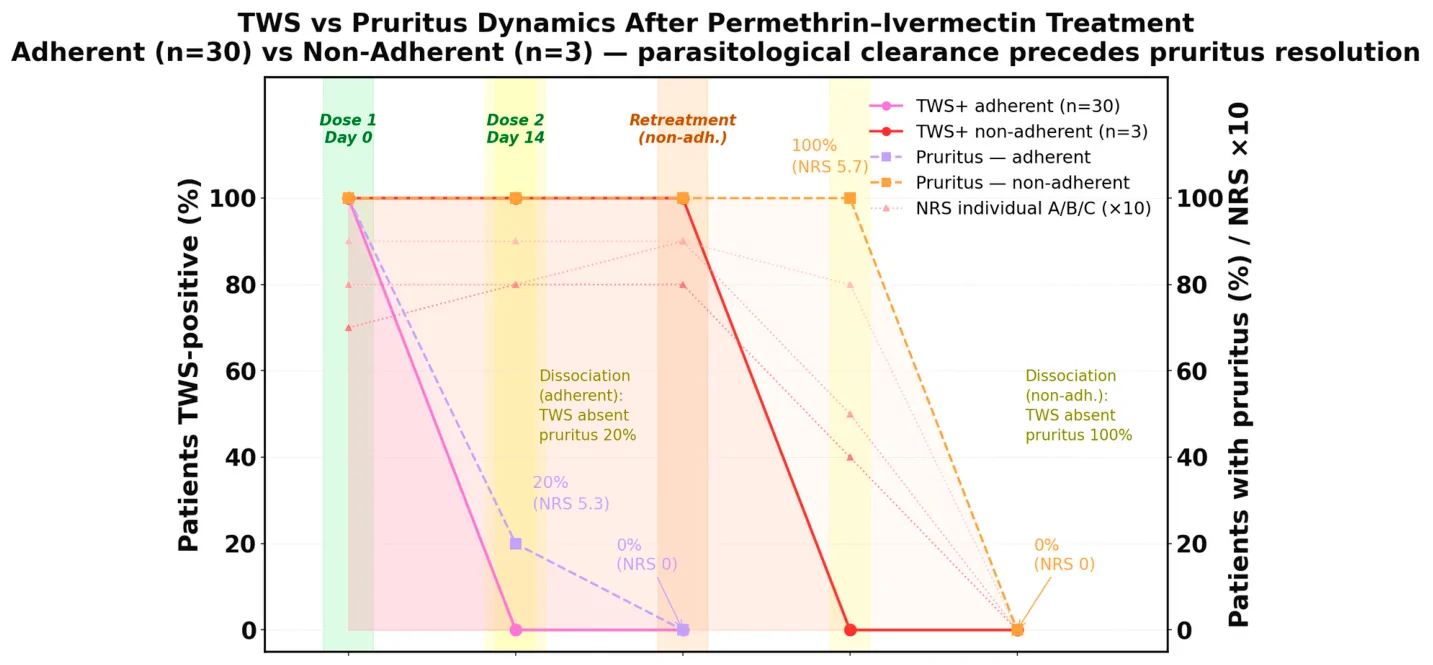
Temporal dynamics of TWS and pruritus by treatment adherence. TWS positivity (solid lines) and pruritus prevalence (dashed lines) over time in adherent (*n* = 30) and non-adherent (*n* = 3) patients. Dotted lines: individual NRS scores for non-adherent patients A, B, C (×10 scaled). Green bands: permethrin–ivermectin doses; orange band: retreatment of non-adherent patients at Week 8. Yellow highlights mark the dissociation timepoints at which TWS had resolved but pruritus persisted.

Of 33 patients enrolled in Cohort 1, three deviated from the prescribed regimen: patients A and B did not take oral ivermectin (cost-related), while patient C took oral ivermectin but omitted topical permethrin and environmental decontamination. Across these 3 patients, 11 burrows were imaged at baseline (4 in patient A, 3 in patient B, 4 in patient C); TWS was detected in 7/11 burrows under polarised dermoscopy and 8/11 under UVFD, consistent with the modality effect observed in the per-protocol cohort (no burrows were polarised-positive only). All 3 patients showed persistence of TWS in ≥1 burrow under UVFD at both the 4- and 8-week reassessments (3/3 at each timepoint), in striking contrast to the complete disappearance of TWS in all 30 adherent patients at the same timepoints. After being re-counselled at week 8, all 3 patients completed the full regimen with concurrent environmental decontamination. At the 12-week reassessment, TWS had disappeared in all 3 patients (0/3 TWS-positive); pruritus, however, lagged behind. NRS scores in patients A/B/C were 7/8/9 at baseline; 8/8/9 at week 4; 8/9/9 at week 8 (paralleling persistent TWS-positivity); 4/5/8 at week 12 (despite TWS clearance); and 0/0/0 at week 16, coinciding with complete clinical resolution. This bifurcation in kinetics between adherent and non-adherent patients (Figure 9) is consistent with TWS resolution tracking clinical clearance rather than time since treatment; given the small subgroup (*n* = 3) and self-reported adherence, these observations are hypothesis-generating, and the within-subgroup TWS–pruritus dissociation (week 12 vs. week 16) mirrors that observed in adherent patients (week 4 vs. week 8).

## 4. Discussion

This study provides the first quantitative validation of the tunnel window sign as a dermoscopic marker of active scabies. Four main findings emerge.

First, UVFD detects TWS more sensitively than polarised dermoscopy at the level of the individual burrow (68/106 vs. 60/106 burrows; McNemar exact *p* = 0.0078) (Figure 3 and Figure 4). This advantage corresponds to eight additional TWS-positive burrows captured by UVFD with no burrows scored positive solely under polarised light and was consistent across all three anatomical sites. This is biologically plausible; at 365 nm, the intact stratum corneum emits only a faint, uniform autofluorescence, whereas keratin-disrupted foci fluoresce bright blue, so the small burrow roof openings may stand out more conspicuously than under polarised light [18]. We propose that, in routine practice, burrows be examined until one TWS-positive burrow is found, which is enough to indicate active scabies. Confirming the absence of TWS, however, requires examining all accessible lesions, since a single TWS-negative burrow does not exclude active scabies.

Second, and more importantly, TWS demonstrates high specificity for active scabies infestation. Across 30 patients with excoriated pruritic dermatoses—including atopic dermatitis, contact dermatitis, and pruritic xerosis—and 150 imaged lesions, not a single TWS-positive structure was identified under either modality (Figure 7). The combined non-active control group of 60 patients (Cohort 2 plus adherent post-treatment scabies) yielded 0/60 false positives. While previously described signs (triangle, ball, jet-contrail) are specific when seen [8,13,14], in practice, their visibility may be reduced by patient scratching and burrow disruption—as may that of TWS itself. Because each burrow was imaged with both modalities, any scratching-related loss of visibility applies equally to polarised dermoscopy and UVFD and therefore does not affect the comparison between them. TWS was detected in all active scabies cases under UVFD (30/30 at the patient level; 68/106 burrows detected at the burrow level), with no false positives in either control group (0/30 pruritic-dermatoses controls and 0/30 post-treatment patients; specificity 30/30 each, 95% CI 88.6–100.0), suggesting it could serve as an additional, robust diagnostic anchor—particularly in the differential diagnosis of pruritus where the parasite cannot be visualised.

Third, TWS resolved completely following successful scabicidal therapy. The disappearance of UVFD-detected TWS in all 30 adherent patients (McNemar exact *p* < 0.0001) preceded complete resolution of pruritus (Figure 8). Further support for the clinically driven kinetics of TWS came from 3 patients with documented non-adherence, in whom TWS persisted throughout weeks 4 and 8 and cleared only at week 12 after retreatment, while pruritus resolved later, being absent at week 16. The bifurcation between adherent and non-adherent kinetics (Figure 9), together with the within-subgroup dissociation between earlier TWS clearance and later pruritus resolution observed in both subgroups, is consistent with TWS reflecting active infestation and its clinical resolution independently of the residual inflammatory response. TWS may thus serve as an earlier, clinically meaningful endpoint of treatment response than clinical examination alone, helping clinicians to distinguish treatment success from residual post-scabies pruritus (a recognised clinical entity [29]) and avoid unnecessary retreatment. Its clinical relevance is heightened by rising rates of permethrin tolerance and treatment failure increasingly reported across Europe [30,31].

Fourth, TWS is a visually reproducible pattern that does not require advanced dermoscopic expertise. The re-scoring of all images by four raters with markedly different levels of clinical training yielded an overall Fleiss’ kappa of 0.872 (95% CI 0.817–0.927)—almost-perfect agreement (Figure 6). Crucially, agreement remained high among the three raters with no role in image acquisition or enrolment (a dermoscopy expert, a novice dermatologist and a non-medical observer; pairwise Cohen’s κ 0.80–0.87), so this reproducibility does not depend on the principal investigator’s readings. This is an unusually robust reproducibility profile for a dermoscopic feature: previous work has shown that inter-rater agreement for many established dermoscopic signs varies widely [32] and is often poor for fine-grained morphological structures even among experts [33]. The consistent recognition of TWS across all training levels supports the use of “tunnel window sign” as an intuitive descriptor and suggests that brief reading-based training is sufficient for reliable identification.

These findings are independent of the ongoing nomenclature debate, including the alternative “ventilation holes” terminology proposed on physiological grounds [34,35,36]. The present study does not aim to adjudicate the underlying biology; rather, it demonstrates that—whatever the physiological substrate—the morphological pattern is reproducibly detectable, more sensitive under UVFD than under polarised light, and discriminative for active infestation.

### 4.1. Limitations

First, this is a single-centre study with a restricted phototype range (Fitzpatrick I–II); validation in skin of colour is required [16,37]. Second, image acquisition was performed by a single operator, which may limit the generalisability of the imaging protocol to other operators and settings. Third, within-patient clustering was substantial (α = 0.40); we accordingly complemented McNemar’s test with a GEE sensitivity analysis, which confirmed the modality effect (OR 1.38, 95% CI 1.07–1.79). The patient-level analyses are independent of this clustering. Fourth, no histopathological or ultrastructural correlation was undertaken; the biological substrate of TWS remains to be elucidated. Fifth, no patient had facial involvement, which is expected: facial scabies is rare in adults and occurs chiefly in young children below the age range of this cohort (youngest patient 14 years). Whether TWS is identifiable on facial scabies in young children—where the anatomical and biological microenvironment differs—remains to be addressed. Sixth, the non-adherent subgroup comprised only three patients whose non-adherence was established by self-report; it precluded formal between-modality testing and was analysed descriptively. These observations are hypothesis-generating rather than confirmatory. Seventh, the principal investigator who enrolled the patients, acquired all images, and originally described the sign could not be blinded to his own diagnostic expectations; although three further independent raters—who had no role in patient enrolment or image acquisition—scored anonymised images in randomised order and agreed closely among themselves, we cannot fully exclude observer bias in the index-test reading. Eighth, no parasitological endpoint (such as microscopy) was obtained after treatment; TWS was therefore validated against a clinical and dermoscopic reference standard, not a parasitological gold standard; magnified UVFD has been proposed to assess mite viability and could be combined with TWS in future studies [38]. Accordingly, throughout this study, “active infestation” denotes the clinically and dermoscopically active disease state—with the mite visualised on dermoscopy at entry (IACS 2020 Level A3 [3])—rather than the parasitologically confirmed presence of mites.

### 4.2. Implications and Future Directions

In clinical settings equipped with a UVFD-capable dermatoscope, detecting TWS—together with established signs—increases the diagnostic yield of dermoscopic examination, particularly when the mite is not directly visualised. In pruritic dermatoses where scabies is a differential, the absence of TWS across multiple lesions has strong negative predictive value in our cohort and may support an alternative diagnosis. TWS may have potential as a non-invasive marker of treatment response. Independent external replication—in additional centres, by independent operators, and across diverse phototypes, with multi-rater scoring—is required before broad clinical adoption.

## 5. Conclusions

In a paired-modality study of 33 patients with active scabies (117 burrows: 106 in 30 adherent patients analysed per protocol, 11 in 3 non-adherent), 30 controls with excoriated pruritic dermatoses (150 lesions), and post-treatment follow-up through week 8 (adherent) or week 16 (non-adherent), UVFD detected the tunnel window sign more sensitively than polarised dermoscopy. TWS discriminated active scabies from pruritic dermatoses and post-treatment skin without false positives in our cohort and resolved with successful therapy—including in non-adherent patients only after adherent retreatment, before pruritus fully resolved. These results support TWS as a sensitive, specific, and dynamic dermoscopic marker of active scabies, with applications for diagnosis and monitoring clinical response to treatment.

## Figures and Tables

**Table 1:** Baseline characteristics of the study cohorts.

Characteristic	Cohort 1—Active Scabies (*n* = 33)	Cohort 2—Pruritic Dermatoses (*n* = 30)
Sex, men/women	19/14	15/15
Age, years (range)	14–75	12–45
Fitzpatrick phototype	I–II	I–II
Treatment adherence	30 adherent/3 non-adherent	Not applicable

## Data Availability

The data that support the findings of this study are available from the corresponding author upon reasonable request. The data are not publicly available due to privacy and ethical restrictions.

## Supplementary Materials

The following supporting information can be downloaded at: https://www.mdpi.com/article/10.3390/jcm15186972/s1, Figure S1: Participant flow diagram; Table S1: SSTROBE checklist for observational (cohort) studies; Table S2: STARD 2015 checklist for diagnostic accuracy studies.
