# Supplementary material for: The Tunnel Window Sign Identifies Active Scabies Infestation: A Validation Study
Source: J Clin Med. 2026 Sep 9;15(18):6972. doi: 10.3390/jcm15186972 (PMC13607227; doi:10.3390/jcm15186972)
Supplement: Supplementary file 1 [file jcm-15-06972-s001.zip › jcm-4506052-supplementary.pdf]

Supplementary Material

The Tunnel Window Sign Identifies Active Scabies Infestation: A Validation Study

Manuscript ID: jcm-4506052

Figure S1. Participant flow diagram.

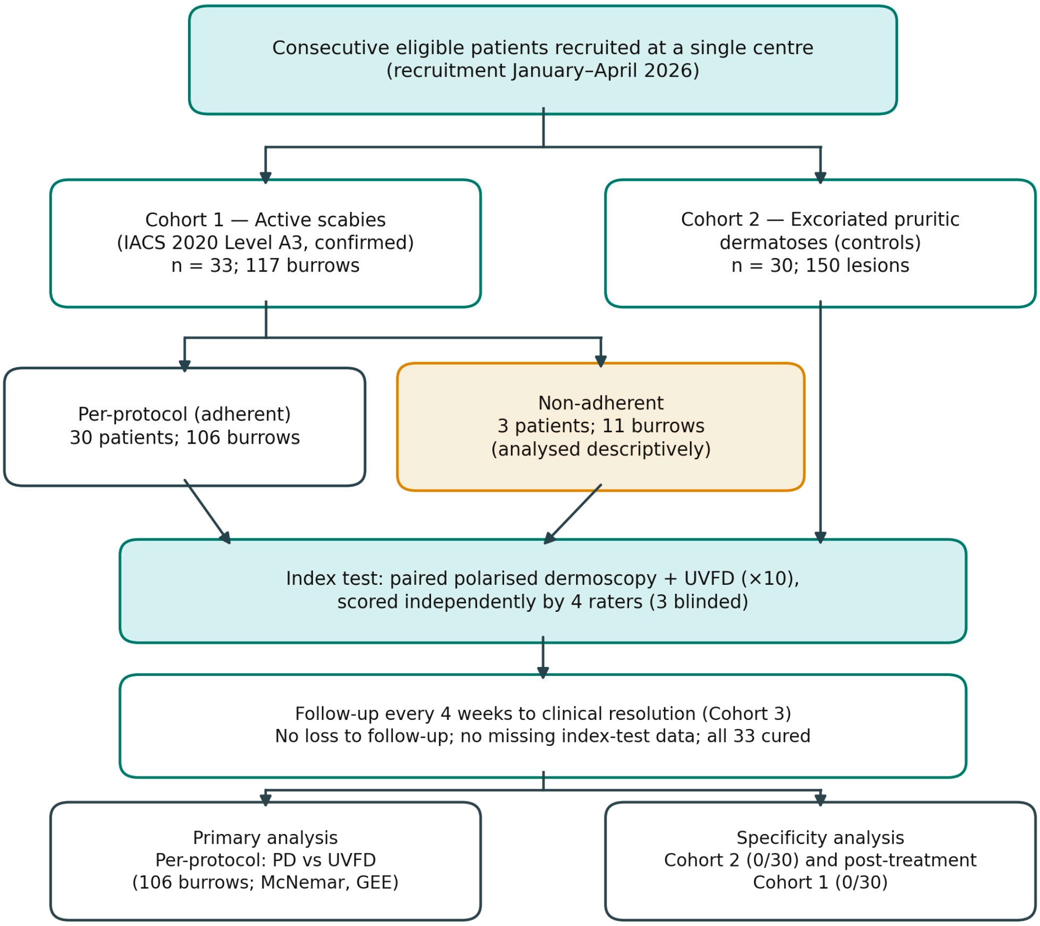

Table S1. STROBE checklist for observational (cohort) studies.

No.	Item — Recommendation	Reported in
1	Title and abstract — (a) study design in title/abstract; (b) balanced summary	Title; Abstract
2	Background/rationale	Section 1

3	Objectives, including any pre-specified hypotheses	Section 1; Abstract
4	Study design — present key elements early	Section 2.1
5	Setting, locations, and relevant dates (recruitment, follow-up)	Section 2.1 (single centre; Jan–Apr 2026)
6	Participants — eligibility criteria, sources, methods of selection	Section 2.2
7	Variables — outcomes, exposures, predictors, effect modifiers	Sections 2.4, 2.5
8	Data sources / measurement for each variable	Sections 2.3, 2.4
9	Bias — efforts to address potential sources of bias	Sections 2.4, 4.1 (index-test blinding; observer bias)
10	Study size — how arrived at	Section 2.1 (consecutive convenience sample; no formal calculation)
11	Quantitative variables — handling; groupings	Section 2.5
12	Statistical methods	Section 2.5
13	Participants — numbers at each stage; flow diagram	Section 3.1; Figure S1
14	Descriptive data — characteristics; missing data	Section 3.1; Table 1 (no missing data)
15	Outcome data	Sections 3.3–3.9
16	Main results — estimates and precision (95% CI)	Sections 3.3, 3.7
17	Other analyses — subgroups (exploratory)	Sections 2.5, 3.4 (characterised as exploratory/post-hoc in Section 2.5)
18	Key results with reference to objectives	Section 4
19	Limitations	Section 4.1
20	Interpretation	Section 4
21	Generalisability	Sections 4.1, 4.2
22	Funding	Funding statement

Table S2. STARD 2015 checklist for diagnostic accuracy studies.

No.	Item	Reported in
1	Identification as a study of diagnostic accuracy (validation)	Title; Abstract

2	Structured abstract	Abstract
3	Scientific and clinical background	Section 1
4	Study objectives and hypotheses	Section 1; Abstract
5	Study design (prospective / retrospective)	Section 2.1 (prospective)
6	Eligibility criteria	Section 2.2
7	How participants were identified	Section 2.2
8	Where and when participants were enrolled (dates)	Section 2.1 (Jan–Apr 2026)
9	Whether consecutive, random or convenience series	Section 2.1 (consecutive)
10a	Index test, in sufficient detail	Sections 2.3, 2.4
10b	Reference standard, in sufficient detail	Section 2.2 (clinical resolution)
11	Rationale for the reference standard	Section 2.2
12a	Definition of index-test positivity (pre-specified?)	Section 2.4 (binary present/absent)
12b	Definition of reference-standard positivity	Section 2.2
13a	Blinding of index-test readers to reference standard	Section 2.4
13b	Blinding of reference-standard assessors to index test	Section 2.2 (clinical, assessed separately)
14	Methods for estimating diagnostic accuracy	Section 2.5
15	How indeterminate results were handled	Section 2.5 (binary; none)
16	How missing data were handled	Section 2.5 (no missing data)
17	Analyses of variability (subgroups)	Sections 2.5, 3.4 (exploratory/post-hoc; characterised in Section 2.5)
18	Intended sample size and how determined	Section 2.1 (no formal calculation)
19	Flow of participants (diagram)	Figure S1
20	Baseline demographic and clinical characteristics	Section 3.1; Table 1
21a	Distribution of disease severity	Section 3.1
21b	Other diagnoses in participants without target condition	Section 2.2 (control diagnoses)
22	Time interval between index test and reference standard	Section 2.3 (paired, same examination)
23	Cross-tabulation of index test vs reference standard	Section 3.3; Figures 3–4
24	Estimates of accuracy and their precision (95% CI)	Section 3.7

25	Adverse events from index test or reference standard	Section 3.1 (none)
26	Study limitations	Section 4.1
27	Implications for practice	Section 4.2
28	Registration number and registry name	Section 2.1 (observational; not registered)
29	Where the full study protocol can be accessed	Section 2.1; Data Availability Statement
30	Sources of funding and role of funders	Funding statement

Section references correspond to the revised manuscript; exact page and line numbers will be finalised on the typeset proof.
